# Prevalence and risk factors of testicular microlithiasis in patients with hypospadias: a retrospective study

**DOI:** 10.1186/s12887-018-1151-6

**Published:** 2018-05-29

**Authors:** Michiko Nakamura, Kimihiko Moriya, Yoko Nishimura, Mutsumi Nishida, Yusuke Kudo, Yukiko Kanno, Takeya Kitta, Masafumi Kon, Nobuo Shinohara

**Affiliations:** 1 Department of Renal and Genitourinary Surgery, Hokkaido University Graduate School of Medicine, North-15, West-7, Kita-Ku, Sapporo, 060-8638 Japan; 2 Diagnostic Center for Sonography, Hokkaido University Hospital, Sapporo, Japan; 3 Division of Laboratory and Transfusion Medicine, Hokkaido University Hospital, Sapporo, Japan

**Keywords:** Testicular microlithiasis, Hypospadias, Undescended testis, Ultrasonography

## Abstract

**Background:**

It has been described that the incidence of testicular microlithiasis is high in several congenital disorders which may be associated with testicular impairment and infertility. Several reports have shown that a prepubertal or pubertal hormonal abnormality in the pituitary-gonadal axis was identified in some patients with hypospadias that is one of the most common disorders of sex development. However, exact prevalence or risk factors of testicular microlithiasis in patients with hypospadias have not reported so far. In the present study, to clarify the prevalence and risk factors of testicular microlithiasis in patients with hypospadias, a retrospective chart review was performed.

**Methods:**

Children with hypospadias who underwent testicular ultrasonography between January 2010 and April 2016 were enrolled in the present study. Severity of hypospadias was divided into mild and severe. The prevalence and risk factors of testicular microlithiasis or classic testicular microlithiasis were examined.

**Results:**

Of 121 children, mild and severe hypospadias were identified in 66 and 55, respectively. Sixteen children had undescended testis. Median age at ultrasonography evaluation was 1.7 years old. Testicular microlithiasis and classic testicular microlithiasis were documented in 17 children (14.0%) and 8 (6.6%), respectively. Logistic regression analysis revealed that presence of undescended testis was only a significant factor for testicular microlithiasis and classic testicular microlithiasis. The prevalence of testicular microlithiasis or classic testicular microlithiasis was significantly higher in children with undescended testis compared to those without undescended testis (testicular microlithiasis; 43.8% versus 9.5% (*p* = 0.002), classic testicular microlithiasis; 37.5% versus 1.9% (*p* < 0.001).

**Conclusions:**

The current study demonstrated that the presence of undescended testis was only a significant risk factor for testicular microlithiasis or classic testicular microlithiasis in patients with hypospadias. As co-existing undescended testis has been reported as a risk factor for testicular dysfunction among patients with hypospadias, the current findings suggest that testicular microlithiasis in children with hypospadias may be associated with impaired testicular function. Conversely, patients with isolated HS seem to have lower risks for testicular impairment. Further investigation with longer follow-up will be needed to clarify these findings.

## Background

Hypospadias (HS) is one of the most common disorders of sex development, occurring in 0.52 to 8.2 of every 1000 live male births [1, 2]. Although the exact etiology of HS is unknown in the majority of patients, a multifactorial etiology including genetic, endocrine and environmental factors is considered to be involved in the genesis of this disorder [3]. HS is also considered as one of the symptoms of testicular dysgenesis syndrome (TDS), which was proposed in 2001 [4]. It has been speculated that impaired development of fetal testes could lead to increased risk of undescended testis (UDT), HS, decreased spermatogenesis and testicular cancer [5]. Several reports have shown that a prepubertal or pubertal hormonal abnormality in the pituitary-gonadal axis was identified in some patients with HS from endocrinological point of view [6–10], which is compatible with the concept of TDS.

Testicular microlithiasis (TM) is characterized by multiple, small, uniform-appearing echogenic foci of less than 3 mm without acoustic shadowing in the seminiferous tubules, which may be indicative of degeneration of the testicular parenchyma [11, 12]. Several theories about the origin or causes of TM have been reported, however, the exact etiology of TM still remains unclear [13]. Previous studies have reported an association between TM and testicular germ cell tumors and/or carcinoma in situ [14, 15]. In addition, an association between TM and infertility has been reported [15]. Although real impact of TM in children is still a matter of debate, it has been described in previous reports that the incidence of TM is high in some congenital disorders, such as UDT, Down’s syndrome, Klinefelter syndrome, McCune-Albright syndrome and Peuzt-Jeghers syndrome, which may be associated with testicular impairment and infertility [16–20].

Based on these previous reports, TM may be a sign of a future endocrinological abnormality in the pituitary-gonadal axis [6–10] or testicular malignancy among patients with HS as a phenotype of TDS. Therefore, we performed testicular ultrasonography (US) for the screening of testicular abnormalities in patients with HS. Because several reports demonstrated that patients with associated genital anomaly, including UDT, were at higher risk for impaired testicular function [6, 9, 21], we speculated that TM may be identified at a higher rate in such patients. However, the exact prevalence of and TM in patients with HS has not been reported so far.

In the present study, we retrospectively examined the prevalence and the risk factors of TM among children with HS.

## Methods

Medical charts of children who visited our hospital for the management or follow-up of HS between January 2010 and April 2016 were retrospectively reviewed. Among them, patients who were born between December 1999 and August 2015, and who underwent on testicular US were included in the present study. Patients with obvious disorders of sex development or with chromosomal abnormalities were excluded. To evaluate and define risk factors of TM in children with HS, the following parameters were assessed with respect to their relation to the prevalence of TM: birth weight, presence of UDT, severity of HS, testosterone administration before HS surgery, and age at US. Severity of HS was divided into mild and severe based on the necessity of transecting urethral plate for correction of chordee deformity according to Koyanagi et al. [22].

**Fig. 1:**
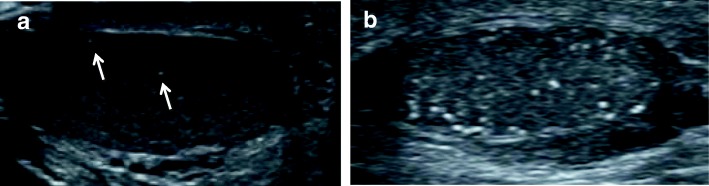
Representative pictures of LTM and CTM. **a** Limited testicular microlithiasis (arrows) in an 11-year-old boy with hypospadias and undescended testis. Ultrasonography of right testis showed 2 small, uniform-appearing echogenic foci without acoustic shadowing. His left testis also displayed 4 echogenic foci per field. **b** Classic testicular microlithiasis in a 1-year-old boy with hypospadias. Ultrasonography of left testis demonstrated more than 5 echogenic foci per field. Right testis also displayed more than 5 echogenic foci per field

TM was defined as 1 or more foci measuring 1 to 3 mm in diameter on testicular US. TM was classified as limited TM (LTM, echogenic foci < 5 /field) or classic TM (CTM, echogenic foci ≥ 5 /field) as reported by Goede et al. [23] (Fig. 1). Among patients with TM, children with CTM in at least one testis were diagnosed with CTM, whereas others were classified as having LTM.

All US evaluation was performed without sedation by sonographers. US evaluations were performed using a PLT-1204BT, a linear probe, 7.2–18 MHz equipped with Aplio™ XG/500 (Toshiba Medical Systems Corp., Tochigi, Japan), a EUP-65, a linear probe 6–14 MHz equipped with HI VISION Avius (Hitachi- Medical, Tokyo, Japan), and a ML6–15, a linear probe 6–15 MHz equipped with Logiq E9 (GE Healthcare, Amersham, UK). If multiple examinations were performed during follow-up, the final assessment findings were evaluated for prevalence and risk factors of TM.

JMP®pro version 12 was used for all statistical analyses. Statistical analyses were performed using logistic regression analysis and Fisher’s exact test. *P* < 0.05 was considered significant.

## Results

Among 219 children who visited during the study period, 121 children (55.3%) were included in the current study. Patient characteristics are shown in Table 1. Median birth weight was 2456 g, and the number of children with low birth weight (less than 2500 g) was 62. Mild and severe HS were identified in 66 and 55 children, respectively. UDT was observed in 16 patients (bilateral in 11, unilateral in 5). Of those, 4 had mild HS, and 12 had severe HS. Regarding co-existing congenital anomaly, inguinal hernia and heart anomaly were observed in 13 and 8, respectively. Testosterone administration before HS surgery was performed in 94 children. Median age at testicular US was 1.7 years old.

**Table 1:** Patient characteristics

	*n* = 121	range
Birth weight (g, median ± SD)	2456 ± 834 (unknown 1)	(472–4048)
Low birth weight (< 2500 g) (pts)	62	
Type of HS (pts)	mild: 66 / severe: 55	
UDT (pts)	16 (unilateral: 5 / bilateral: 11)	
Testosterone administration before surgery (pts)	yes: 94, no: 20 (unknown 7)	
	topical: 67 / systemic: 10 / topical+systemic: 17	
Age at USG (yrs, median ± SD)	1.7 ± 4.0	(0.5–18.2)
TM (pts) (LTM / CTM)	17 (9 / 8)	

### Prevalence of TM and CTM

TM and CTM were documented in 17 children (14.0%) and in 8 children (6.6%), respectively. TM was identified unilaterally in 7, and bilaterally in 10. Among 8 children with CTM, bilateral CTM was identified in 5. In the remaining 3 children, unilateral CTM alone was detected in 1, and CTM on one side and LTM on the other side in 2. No testicular tumors were detected on US in any children.

### Risk factors of TM and CTM

Univariate analysis demonstrated that the severe type of HS and presence of UDT were risk factors for TM. On multivariate analyses, presence of UDT was only a risk factor for TM (*p* = 0.006) (Table 2). Regarding CTM, presence of UDT was only a risk factor on univariate analysis (*p* < 0.001) (Table 3). The incidence of TM or CTM was significantly higher in children with UDT compared with those without UDT (TM; 43.8% versus 9.5% (*p* = 0.002), CTM; 37.5% versus 1.9% (*p* < 0.001)) (Table 4).

**Table 2:** Risk factors for TM

	Univariate analysis		Multivariate analysis	
	Odds ratio (95% confidence interval)	*p* value	Odds ratio (95% confidence interval)	*p*-value
Birth weight (g, median ± SD)	0.44 (0.04–4.09)	n.s.		
Low birth weight (< 2500 g)	0.84 (0.68–4.09)	n.s.		
Severe type of HS	3.40 (1.17–11.35)	0.024^*^	2.48 (0.79–8.65)	n.s.
UDT	7.39 (2.23–24.55)	0.001^*^	5.8 (1.68–19.95)	0.006^*^
Testosterone administration	1.57 (0.39–10.59)	n.s.		
Age at USG	0.28 (0.04–2.52)	n.s.		

^*^p < 0.05

**Table 3:** Risk factors for CTM

	Univariate analysis	
	Odds ratio (95% confidence interval)	*p*-value
Birth weight (g, median ± SD)	4.26 (0.20–85.70)	n.s.
Low birth weight (< 2500 g)	1.61 (0.38–8.14)	n.s.
Severe type of HS	3.92 (0.86–27.53)	n.s.
UDT	30.90 (6.23–231.37)	< 0.001^*^
Age at USG	0.19 (0.01–3.62)	n.s.

^*^p < 0.05

**Table 4:** Prevalence of TM and CTM

	UDT (+)	UDT (−)	*p*-value
Prevalence of TM	43.8% (7/16)	9.5% (10/105)	0.002
Prevalence of CTM	37.5% (6/16)	1.9% (2/105)	< 0.001

### Side of TM and UDT (Table 5)

In 11 children with bilateral UDT, unilateral and bilateral TM were observed in 1 and 4, respectively. Among them, CTM in at least one side was identified in 4. Of 5 with unilateral UDT, TM was identified in 2 (both with bilateral CTM). Among 105 children without UDT, unilateral and bilateral TM were observed in 6 and 4, respectively. Of those, CTM in at least one side was observed only in 2.

**Table 5:** Sides of testicular microlithiasis and undescended testis

	Bilateral UDT	Unilateral UDT	Without UDT
	*n* = 11	*n* = 5	*n* = 105
Unilateral TM	1	0	6
CTM	1	0	0
LTM	0	0	6
Bilateral TM	4	2	4
Bilateral CTM:	2	2	1
CTM and LTM	1	0	1
Bilateral LTM	1	0	2

## Discussion

To our knowledge, the current study represented the first report on the prevalence of TM in children with HS. TM and CTM were identified in 14.0 and 6.6%, respectively, of children with HS. Presence of UDT was only a risk factor for TM and CTM.

Previous studies have shown that TM is associated with several conditions, including impaired spermatogenesis, testicular cancer and carcinoma in situ [14, 15]. In asymptomatic adults, the rate of CTM varies from 0.6 to 9% [24, 25]. Recent studies revealed that the prevalence of TM and CTM in asymptomatic boys was 4.2 and 2.4% respectively, and increased with age [23]. The prevalence of TM (14.0%) and CTM (6.6%) in children with HS in the current study seems to be relatively higher compared with that in asymptomatic boys in the previous reports. Although the etiology of HS is considered to be multifactorial, the concept of TDS, which suggests that impaired development of fetal testes could lead to increased risk of HS, has been proposed as one of the causes of HS. Drut et al. proposed that TM may be related with Sertoli cell dysfunction and abnormal embryogenesis during the early stages of testicular development [12]. Wohlfahrt-Veje et al. reported that dysgenetic testes often have an irregular US pattern in which TM may also be visible [5]. The reason for the relatively high prevalence of TM may be due to that children who have such embryological causes of testis anomaly could have been included in the present study.

We demonstrated that the presence of UDT was only a risk factor for TM and CTM. There are several reports of prepubertal or pubertal hormonal abnormalities of the pituitary-gonadal axis in some patients with HS [6–10]. In addition, there are several reports on patients with both HS and UDT, which is a risk factor of TM and CTM as demonstrated in our study, who were at a higher risk for decreased testicular function or impaired spermatogenesis [6, 21]. A number of reports have demonstrated the relationship between TM and impaired spermatogenesis [26–28], although this issue is still controversial [29]. Accordingly, TM in children with HS may be associated with decreased testicular function and/or impaired spermatogenesis. To determine the relationship between TM and testicular function/spermatogenesis in patients with HS, further follow-up with endocrinological evaluations until puberty is necessary.

While the prevalence of TM in patients with HS and without UDT (9.5%) in the current study was slightly higher compared to that in asymptomatic boys (4.2%) reported in the previous literature [23], the prevalence of CTM (1.9%) was almost similar to that in asymptomatic boys (2.4%). Accordingly, patients with isolated HS seem to have lower risks for testicular impairment. On the contrary, although the presence of UDT in patients with HS was demonstrated as a risk factor for TM in the current study or impaired semen quality in the previous report [21], there has been no comparative study focusing on risk of TM or testicular dysfunction between patients with isolated UDT and those with HS and UDT. To clarify the impact of HS in patients with UDT in terms of the risk of TM or testicular dysfunction, additional studies are necessary.

Previous studies reported that the prevalence of primary testicular tumors in patients with TM ranged from 15 to 45% [15, 29]. Thus, there was some concern that TM may lead to testicular cancer at the end of the 1990’s. However, two studies revealed that the rate of TM in the asymptomatic population ranged from 2.4 to 5.6% [24, 25], which is much higher than the prevalence of the lifetime risk of testicular cancer in the general population. Nowadays, it is recognized that TM in adults without known risk factors, such as previous testicular cancer, a history of UDT or testicular atrophy, seems to be a benign condition [19, 24, 25, 30]. Regarding TM detected in childhood, Suominen et al. found 15 patients with neoplasms among 421 pediatric patients (3.6%) by systematic review [31]. They described that TM should be considered a benign condition even in the pediatric age group, but the fact that TM is associated with testicular malignancy (< 5%) cannot be ignored. Although the concept of TDS included symptoms of HS, UDT and testicular cancer [5], as far as we know, there is no report demonstrating that the prevalence of testicular tumors is higher in patients with HS. Although UDT is well-known as a risk factor for testicular malignancy [32], it is obscure whether UDT is also a risk factor for testicular malignancy among patients with HS. Longer follow-up will clarify the exact associations among testicular malignancy, TM and/or UDT in children with HS.

There is some controversy regarding the method and duration of follow-up in patients with TM. In the guidelines produced by the European Society of Urogenital Radiology, the consensus opinion is that the presence of TM alone in the absence of other risk factors is not an indication for regular follow-up in adults [19]. However, this guideline did not mentioned children with HS. At this time, we believe that the follow-up protocol for patients with HS and TM should be determined based on the presence or absence of UDT because the exact risk for testicular malignancy in patients with HS alone remains obscure.

Several limitations of the present study should be addressed. First, this study was conducted in a retrospective nature and relatively small number of children. Second, there was no control group such as Japanese boys who had no genital disease or isolated UDT. Third, evaluation of chromosomal abnormalities was not performed in all children. Fourth, as children included in the current study were relatively young and because TM sometimes appears later in childhood [23], the true prevalence of TM in patients with HS may be higher than the prevalence in this study. Fifth, as endocrinological examination or semen analysis was not performed in the current study, testicular function could not be compared between patients with and without TM.

## Conclusions

TM and CTM were identified in roughly 14.0 and 6.6% of children with HS, respectively. The prevalence of TM and CTM was significantly higher in patients with UDT. As UDT among children with HS has been reported as a risk factor for endocrinological abnormality and/or impaired spermatogenesis, these findings suggest that TM in children with HS may be associated with impaired testicular function. In addition, the prevalence of CTM in patients with isolated HS was almost equal to the previously reported prevalence in asymptomatic boys. Therefore, patients with isolated HS seem to have lower risks for testicular impairment. Further investigation with longer follow-up will be needed to clarify these findings.

## Abbreviations

CTM: Classic testicular microlithiasis
HS: Hypospadias
LTM: Limited testicular microlithiasis
TDS: Testicular dysgenesis syndrome
TM: Testicular microlithiasis
UDT: Undescended testis
USG: Ultrasonography
